# Acknowledgement to Reviewers of *Plants* in 2017

**DOI:** 10.3390/plants7010005

**Published:** 2018-01-12

**Authors:** Plants Editorial Office

**Affiliations:** MDPI AG, St. Alban-Anlage 66, 4052 Basel, Switzerland; plants@mdpi.com

Peer review is an essential part in the publication process, ensuring that *Plants* maintains high quality standards for its published papers. In 2017, a total of 63 papers were published in the journal. Thanks to the cooperation of our reviewers, the median time to first decision was 20 days and the median time to publication was 48 days. The editors would like to express their sincere gratitude to the following reviewers for their time and dedication in 2017:

Antonio, Amilcar L.Kumagai, EtsushiAparicio, Pedro MKumar, AshutoshArtyszak, ArkadiuszKunst, LjerkaAtes, SerkanLatosińska, JolantaAugust-Schmidt, Elizabeth M.Lee, Sang-HanBayer, EmmanuelleLeone, AlessandroBazos, IoannisLi, WanlongBenedec, DanielaLidon, FernandoBenitez-Alfonso, YoselinLin, Ming-KuemBerhow, MarkLiu, ChengwuBlack, Katherine A.Lizamore, DarrellBobis, OtiliaLopez-Moya, FedericoBörner, AndreasMa, WuBoss, PaulMacnack, NatashaBotta, BrunoMarchetti, MartaBožović, MijatMarchini, CristinaBravo, LeónMarcovici, GenoBroughton, Katrina J.Martínez, SidoniaBuchet, ReneMartins, Maria RosarioBurcul, FrankoMathesius, UlrikeBurkey, Kent O.Mattei, BenedettaBussmann, Rainer W. Mcewan, RyanCádiz Gurrea, María De La LuzMedina, JoaquínCapasso, RaffaeleMelkonyan, AniCardoso, SusanaMenand, BenoîtCaruso, GiuseppeMenendez, EstherCazzato, EugenioMoriguchi, TakayaChaterjee, AmitavaMorkunas, IwonaChizzola, RemigiusMurata, ToshihiroCho, Tae-JuNakagawa, TsuyoshiChristensen, LarsNawrath, ChristianeContreras, MaríaNiemeyer, Emily D.Ćosić, JasenkaO’shaughnessy, SusanDapprich, PeterOshima, YoshimiDe La Torre, FernandoPalla, FrancoDebener, ThomasPalmgreen, Michael G.Del Giudice, RitaPanday, DineshDeligios, Paola A.Parsonage, DerekDiago, MPPatra, BarunavaDomergue, FredericPellegrini, ElisaDomozych, David S.Plummer, SarahDosoky, Noura S.Qualset, Calvin ODriscoll, DonReed, KevinDuarte, Maria CristinaResende, Marcio FRDugé De Bernonville, ThomasRestivo, Francesco MariaDurazzo, AlessandraRibeiro-Barros, Ana I.Eiroa, José LuisRivero, RosaEl Babili, FatihaSalazar-Olivo, LuisElhanafi, Ahmad OmarSaleem, MuhammadEmani, ChandrakanthSalehi-Ashtiani, KouroshEndresen, DagSalehin, MohammadEngelberth, JurgenSarnowski, TomaszEspen, LucaSawicki, RafalEudes, AymerickSchinkovitz, AndreasFernandez-Ondoño, EmiliaSchneitz, KayFreitas, MarisaSchwarzkopf, LinFrew, AdamScognamiglio, MonicaGarcia, Lorena CarroScotti Campos, PaulaGeilfus, Christoph-MartinSelim, SamehGerm, MatejaSerrano, MaríaGiannakoula, AnastasiaSgherri, CristinaGiovannini, AnnalisaShoeva, Olesya YuGniewosz, MałgorzataShrestha, Krishna KumarGolubkina, Nadezda AleksandrovnaSingh, ShardenduGonzález-Stuart, ArmandoSingh-Cundy, AnuGranada, Camille E.Smiesko, MartinGreene, DuaneSobeh, MansourGreer, MitchellSpampinato, ClaudiaGrossnickle, Steven C.Speciale, AntonioGruber, ChristianSpies, JanineGuinel, Frédérique C.Sroka, ZbigniewGulati, VandanaStavolone, LiviaGullón, BeatrizStout, Michael J.Guy, Charles L.Taira, JunseiHajimorad, RezaTakeda, SeijiHammerschmidt, RayTellez, Trinidad RuízHannapel, David J.Thilmony, RogerHano, ChristopheTirillini, BrunoHatzopoulos, PolydefkisTombesi, SergioHaughian, Sean R.Torricelli, RenzoHelariutta, YrjöTsiani, EvangeliaHildreth, SherryTytła, MalwinaHodson, Martin JUhde-Stone, ClaudiaHögbom, LarsUlery, AprilHolalu, SrinidhiVaknin, YiftachHorgan, FinbarrVan Der Linden, GerardHornyik, CsabaVenäläinen, SallaInazumi, ShinyaVialet-Chabrand, SilvereIslam, M. NurulVoitsekhovskaja, Olga V.Janssen, DirkWang, ZhijunJaradat, NidalWesolowski, MarekJarret, Robert L.Wilson, HoustonKacaniova, MiroslavaWojtyla, ŁukaszKasel, SabineWu, GuangxiKhatodia, SurenderXu, WenjiaKieliszek, MarekYasir, IqbalKikuzaki, HiroeYeh, Ching-HuiKnežević, Sanda VladimirYu, Tien-ShinKosma, DylanZhang, HengyouKozaki, AkikoZhao, MeixiaKrishnan, SanalkumarZiebell, HeikoKulus, Dariusz


